# Use of the *HPRT* gene to study nuclease-induced DNA double-strand break repair

**DOI:** 10.1093/hmg/ddv409

**Published:** 2015-09-30

**Authors:** Polly Gravells, Sara Ahrabi, Rajani K. Vangala, Kazunori Tomita, James T. Brash, Lena A. Brustle, Christopher Chung, Julia M. Hong, Aikaterini Kaloudi, Timothy C. Humphrey, Andrew C.G. Porter

**Affiliations:** 1 Gene Targeting Group, Centre for Haematology, Imperial College Faculty of Medicine, London W120NN, UK and; 2 CRUK MRC Oxford Institute for Radiation Oncology, Department of Oncology, University of Oxford, Oxford OX3 7DQ, UK

## Abstract

Understanding the mechanisms of chromosomal double-strand break repair (DSBR) provides insight into genome instability, oncogenesis and genome engineering, including disease gene correction. Research into DSBR exploits rare-cutting endonucleases to cleave exogenous reporter constructs integrated into the genome. Multiple reporter constructs have been developed to detect various DSBR pathways. Here, using a single endogenous reporter gene, the X-chromosomal disease gene encoding hypoxanthine phosphoribosyltransferase (*HPRT*), we monitor the relative utilization of three DSBR pathways following cleavage by *I-Sce*I or CRISPR/Cas9 nucleases. For *I-Sce*I, our estimated frequencies of accurate or mutagenic non-homologous end-joining and gene correction by homologous recombination are 4.1, 1.5 and 0.16%, respectively. Unexpectedly, *I-Sce*I and Cas9 induced markedly different DSBR profiles. Also, using an *I-Sce*I-sensitive *HPRT* minigene, we show that gene correction is more efficient when using long double-stranded DNA than single- or double-stranded oligonucleotides. Finally, using both endogenous *HPRT* and exogenous reporters, we validate novel cell cycle phase-specific *I-Sce*I derivatives for investigating cell cycle variations in DSBR. The results obtained using these novel approaches provide new insights into template design for gene correction and the relationships between multiple DSBR pathways at a single endogenous disease gene.

## Introduction

Inappropriate repair of chromosomal DNA double-strand breaks (DSBs) can lead to mutagenesis, gross chromosomal instability and genetic disease (1). Knowledge of DSB repair pathways, and their defects in particular cancers, has helped to define oncogenic mechanisms and to develop new therapeutic strategies (2). Such knowledge also underpins powerful genome engineering methods that use customized endonucleases, including clustered regulatory interspaced short palindromic repeat (CRISPR)/Cas-based RNA-guided nucleases, to make targeted chromosomal DSBs (3). Therapeutic gene targeting (GT) (4) for the correction of disease-causing mutations is an emerging application of such methods (5).

There are two main DSB repair (DSBR) mechanisms: homologous recombination (HR) (6) and non-homologous end-joining (NHEJ) (7). HR requires a homologous DNA repair template, usually in the form of a sister chromatid, which ensures that the original DNA sequence is restored. Repair templates can be engineered, however, and introduced into cells to make a defined genomic modification via HR (GT). HR requires extensive 5′–3′ end-resection at DSBs to generate single-stranded tails that form a RAD51 nucleofilament capable of invading a duplex DNA template. Requirements for a sister chromatid template and cyclin-dependent kinase activity (8) restrict HR to the S and G2 phases of the cell cycle (9). NHEJ lacks these requirements and can occur throughout the cell cycle. In classical NHEJ, DNA ends are protected by the Ku heterodimer; this minimizes end-resection allowing for accurate end-joining (accNHEJ, also termed precise ligation) (7,10). Here, we use the term accNHEJ to describe the joining of any two DNA ends that have undergone no gain or loss of nucleotides. In some circumstances, such as the absence of Ku heterodimer, NHEJ is more likely to involve limited end-resection, resulting in insertions and/or deletions (indels) at the site of the DSB. Such mutagenic NHEJ (mutNHEJ) often involves the annealing of short (e.g. 2–6 nt) sequence homologies from each side of the DSB (micro-homology-mediated end-joining; MMEJ). In an analogous pathway termed single-strand annealing (SSA), that can also be considered a form of non-conservative RAD51-independent HR, deletions result from extensive end-resection followed by annealing of longer (e.g. >100 nt) repeats (6).

Customized endonucleases can be introduced into cells to generate targeted indels via mutNHEJ or to promote specific modifications via GT. An ability to promote one DSBR pathway at the expense of others would be valuable for facilitating the desired genome engineering outcome. DNA resection, cell cycle phase, DNA end-structure and host cell type are all known to influence DSBR pathway choice (11,12). Further investigations into how these and other variables affect pathway choice will be important to facilitate genome manipulation and improve our understanding of DSBR and genome instability.

Research into DSBR has benefitted greatly from assays for the repair of defined chromosomal DSBs generated by rare-cutting endonucleases. Typically, a homing endonuclease, such as *I-Sce*I, is used to cleave an exogenous reporter construct that has been randomly inserted into the genome. Many such constructs have been developed using reporters that fluoresce or confer drug resistance. Most are designed to detect, without recourse to structural analyses of the repaired locus, a single type of DSBR, including intrachromosomal HR (13,14), GT (15), SSA (16), mutNHEJ or accNHEJ (10,17–20) and MMEJ (21) (reviewed in 22). Simultaneous reporting for multiple DSBR pathways can be achieved by integrating multiple constructs into a single clone (20,23,24) or by integrating a single reporter construct that detects multiple types of repair (25,26).

DSBR mechanisms are known to be affected by chromosomal location (27–31) and by epigenetic factors (32–34) that are in turn affected by exogenous DNA sequences (35–37). It is, therefore, important to minimize or eliminate these variables when comparing different exogenous DSBR reporters. Differences in chromosomal location have been avoided by integrating different constructs at the same locus (16) or mitigated by averaging the behaviour of constructs at multiple integration sites (23). These precautions are rarely taken, however, and do not control for any differences between constructs in their effects on the host genome. For these reasons, single reporters that can detect multiple types of DSBR at a single locus are highly desirable. To date, the only reporters that achieve this without the need to sequence breakpoints are traffic light reporter (TLR) systems that elegantly combine multiple fluorescent marker genes to detect mutNHEJ and GT (25) or mutNHEJ, GT and SSA (26).

In the present study, we use an endogenous gene as a reporter to detect three types of nuclease-induced DSBR: mutNHEJ, GT and accNHEJ. Our reporter is the highly conserved X-chromosomal gene encoding hypoxanthine phosphoribosyltransferase (HPRT), an enzyme that is essential for the salvage pathway of purine biosynthesis, and deficiencies of which cause a range of clinical phenotypes, including Lesch Nyan disease (38). We chose *HPRT* not only because it is a model disease gene, naturally residing and expressed in growing cells, but also because it is drug selectable. Thus, cells with inactive or active *HPRT* alleles can be selected, respectively, in medium containing 6-thioguanine (6TG) or hypoxanthine, aminopterin and thymidine (HAT). Although *HPRT* has to be used extensively for studies of random mutagenesis and un-induced GT (39–43), it has not been systematically exploited for monitoring nuclease-induced DSBR. Here, we cleave *HPRT* alleles with *I-Sce*I or Cas9 nuclease and estimate the relative frequency of three different DSBR outcomes. We also use an *I-Sce*I-sensitive *HPRT* minigene to investigate how the frequency of gene correction is influenced by repair template design. As an additional tool, to facilitate studies of cell cycle control of DSBR, we describe novel *I-Sce*I derivatives that are expressed in a cell cycle-dependent fashion.

## Results

### Silent introduction of an *I-Sce*I site into a mouse *HPRT* minigene

**Figure 1.**
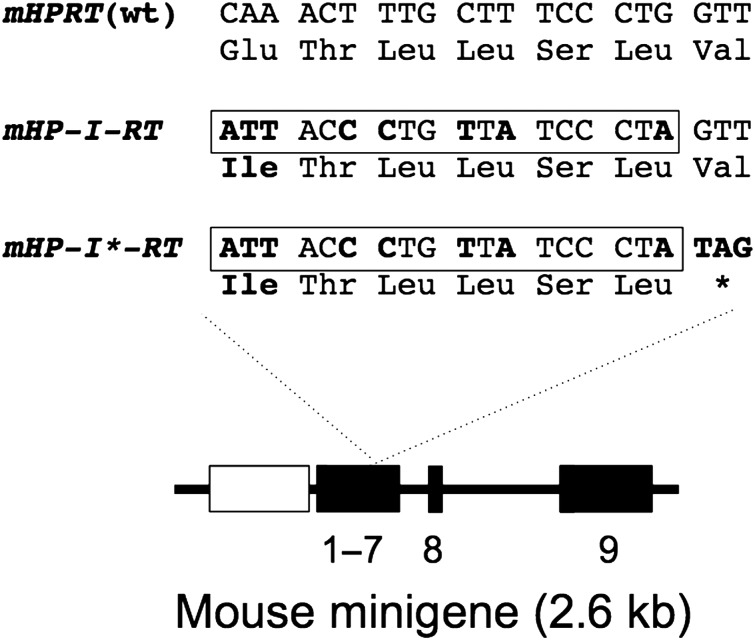
Mouse *HPRT* minigenes used in this study. Numbered black boxes represent exons. The mouse minigene is driven by the PGK promoter (grey box) and all but the last two introns are removed. Partial nucleotide, and amino-acid, sequences from exon 6 are shown for the wild-type (WT) allele and for alleles modified to carry an *I-Sce*I site (boxed) with (mHP-I*-RT) or without (m*HP-I-RT*) an adjacent stop codon. Altered residues are shown in bold.

The *I-Sce*I recognition sequence (ATTACCCTGTTATCCCTA) translates into an amino-acid sequence (ITLLSL) with only one difference to a sequence (QTLLSL) in the normal HPRT enzyme. Assuming this change (Q144I) does not disrupt HPRT function, we introduced the *I-Sce*I site into the exon-6-derived region of a mouse *HPRT* minigene (*mHPRT*) to generate *mHP-I-RT* (Fig. 1). As a control, we introduced a stop codon (*) immediately downstream of the *I-Sce*I site, to generate mHP-I*-RT. When transfected into human *HPRT*^−^ human fibrosarcoma (HT1080) cells, *mHPRT* and *mHP-I-RT* conferred resistance to HAT medium (HAT^R^) equally well, whereas mHP-I*-RT conferred no resistance (Table 1). This indicates that the Q145I mutation has little or no effect on HPRT function, which is consistent with structural and evolutionary considerations (Supplementary Material, Fig. S1).

**Table 1.** Rescue of HPRT^−^ cells with mHP-I-RT

Transfected plasmid	Encoded minigene	Experiment 1^a^	Experiment 2	Experiment 3
pBT/PGK-HPRT (R1)	*mHPRT (wt)*	189	241	266
pBT/PGK-HP-I-RT (R1)	*mHP-I-RT*	198	213	259
pBT/PGK-HP-I*-RT (R1)	mHP-I*-RT	0	0	0

^a^In each experiment, plasmids expressing the indicated minigene were individually electroporated into 1 million HPRT^−^ cells which were then selected in HAT medium to generate the indicated numbers of HAT^R^ colonies.

### *I-Sce*I-induced correction and mutagenesis of a mouse *HPRT* minigene

**Figure 2.**
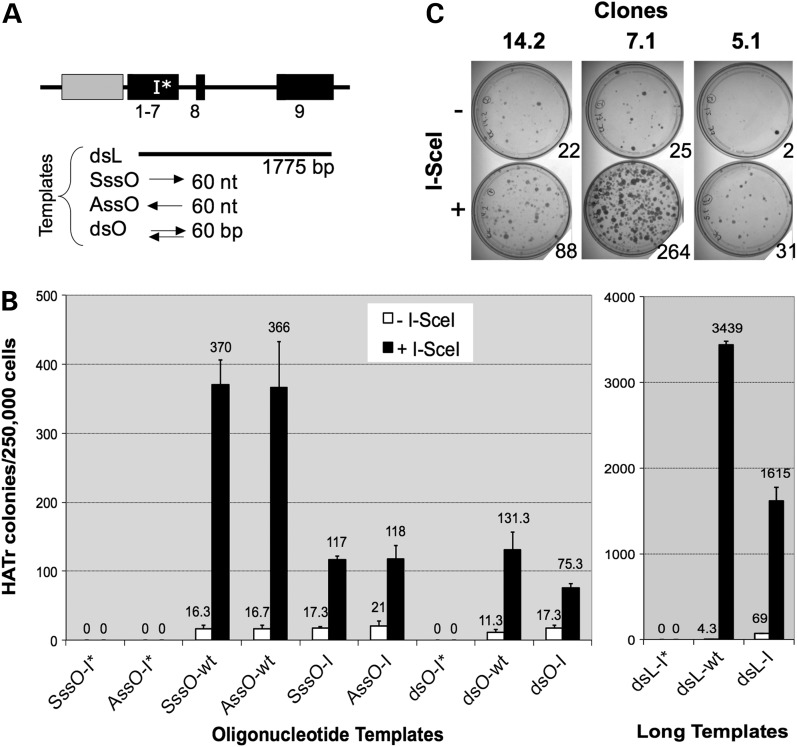
*I-Sce*I-induced correction (**A** and **B**) or mutagenesis (**C**) of an integrated *mHPRT* minigene. (A) Gene correction templates are shown schematically, aligned with the target mHP-I*-RT minigene. Templates were sense or antisense single-stranded oligonucleotides (SssO, AssO), dsOs or dsL (for oligonucleotide sequences, see Supplementary Material, Table S3). Each of these four template classes was made with the *I-Sce*I site and stop codon (-I*), the *I-Sce*I site only (-I) or neither (-wt; wild-type). (B) *HPRT* ^−^ cells (clone 2.1) carrying the mHP-I*-RT minigene and a tetracycline-regulated *I-Sce*I expression cassette were grown with or without tetracycline and transfected with the indicated templates detailed in (A). The frequencies of resulting HAT-resistant colonies are shown for oligonucleotides templates (left panel) and dsL templates (right panel). Error bars show SDs for three experiments. (C) Three HPRT+ clones (14.2, 7.1 and 5.1) carrying the *mHP-I-RT* minigene were transfected with *I-Sce*I expression plasmid (+), or a vector control (−), and selected in 6TG. Colonies resulting from selection of 5 × 10^5^ cells are shown.

To study *I-Sce*I-induced gene correction by HR, the mHP-I*-RT minigene was stably integrated into an *HPRT*^−^ cell line (HTtetSCE; Supplementary Material, Table S1) carrying a tetracycline-regulated *I-Sce*I gene. One of the resulting clones with a single copy of mHP-I*-RT (clone m2.1) grew normally with or without tetracycline. Using this clone, we examined the efficiency of *mHPRT* gene correction by various templates: single- or double-stranded oligonucleotides or long double-stranded DNA (Fig. 2A). Each template had the wild-type sequence, an *I-Sce*I site, or an *I-Sce*I site and adjacent stop codon. Gene correction data are summarized (Fig. 2B) for oligonucleotide templates (left panel) and long templates (right panel). As expected, only templates without the stop codon gave rise to HAT^R^ colonies. Consistent with previous studies (15,44,45), correction by all repair templates was greatly stimulated by *I-Sce*I (up to 800-fold). We also made the following observations (see Discussion for interpretations). First, sense and antisense oligonucleotides supported gene correction equally well. Second, single-stranded oligonucleotides were more effective than double-stranded oligonucleotides. Third, unlike correction with single-stranded templates, where increasing the homology length above 40 nt had little impact (46), increasing the homology length of double-stranded templates from 60 bp to 1.7 kb resulted in a 21- to 26-fold increase in gene correction [Fig. 2B; compare double-stranded oligonucleotide (dsO) and long double-stranded DNA (dsL)]. Fourth, despite having eight additional mismatches to the target, wild-type repair templates were always more effective than their equivalent templates with *I-Sce*I sites. Finally, correction by long double-stranded templates was 9- to 26-fold more efficient than by oligonucleotides.

We next attempted to measure DSB-induced loss of *HPRT* function using clones with integrated *mHP-I-RT* minigenes. Three such HAT^R^ clones (14.2, 7.1 and 5.1; Supplementary Material, Table S1) were transiently transfected with *I-Sce*I expression plasmid (pCMV3xnls-*I-Sce*I) and selected in 6TG. For each clone, *I-Sce*I expression clearly increased the frequency of 6TG-resistant (6TG^R^) colonies (Fig. 2C), but frequencies varied greatly between clones. Such variations, as well as the appreciable frequencies of spontaneous 6TG^R^ colonies, are most likely result of chromosomal position effects.

### Strategy for generating an *I-Sce*I-sensitive human *HPRT^+^* allele

**Figure 3.**
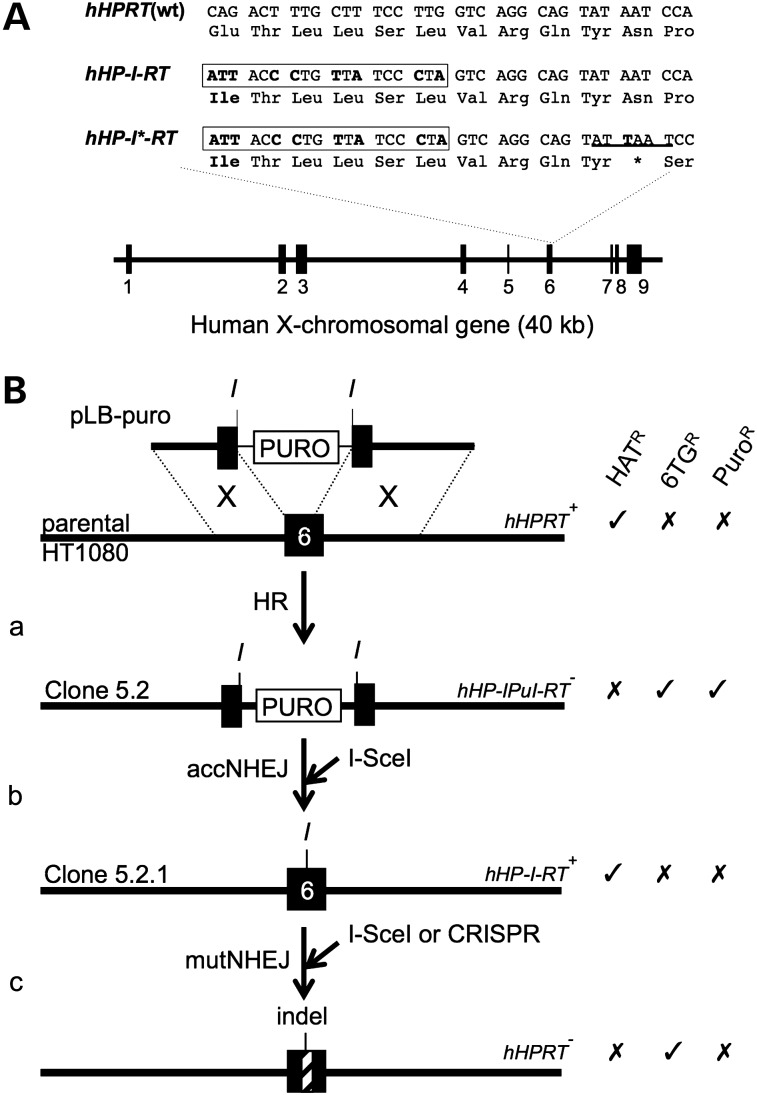
Generating and using *I-Sce*I-sensitive *hHPRT* alleles. (**A**) Structure of parental and *I-Sce*I-sensitive *hHPRT* alleles. Numbered black boxes represent exons. Partial nucleotide and amino-acid sequences for exon 6 are shown for the WT allele and for alleles modified to carry an *I-Sce*I site (boxed), with (hHP-I*-RT) or without (*hHP-I-RT*) an adjacent stop codon. Altered residues are shown in bold. An *Ase*I site in hHP-I*-RT is underlined. (**B**) Strategy for generating *I-Sce*I-sensitive alleles and using them for accNHEJ and mutNHEJ assays. (**a**) Disruption of exon 6 by GT with pLB-puro. (**b**) Basis of accNHEJ assay. An *I-Sce*I-induced DSB repaired by accNHEJ generates a functional *I-Sce*I-sensitive allele. The frequency of resulting HAT^R^ colonies measures accNHEJ. (**c**) Basis of mutNHEJ assay. An *I-Sce*I-induced DSB is repaired by mutNHEJ to generate an indel (hatched box). The frequency of resulting 6TG^R^ colonies measures mutNHEJ. Exon 6 (black box) and adjacent intronic DNA of the chromosomal *HPRT* locus (long lines) are shown. Targeting construct pLB-puro is shown with its puromycin-resistance cassette (PURO, white box) and its 2.7 and 3.1 kb arms aligned with homologous chromosomal regions (dotted lines) to allow HR (X). Sites for *I-Sce*I (*I*) are shown. Alleles are labelled with their names (right) and the names of representative host cell lines (left). Resistance (✓) or sensitivity (✗) to key selective agents is indicated.

To avoid chromosomal position effects and establish a standard endogenous locus for analysing DSBR, we generated *I-Sce*I-sensitive human *HPRT* alleles. The structure of the endogenous *HPRT* gene and *I-Sce*I-sensitive derivatives is shown in Figure 3A. Our strategy for generating these and using them for accNHEJ and mutNHEJ assays is outlined in Figure 3B. First, a targeting construct (pLB-puro) was used to introduce a puromycin-resistance cassette into exon 6 (Fig. 3Ba). This cassette was flanked by *I-Sce*I sites, allowing it to be excised by *I-Sce*I. If excision is followed by accNHEJ of the two resulting chromosome ends, a functional *HPRT* allele carrying an *I-Sce*I site in exon 6 is generated (Fig. 3Bb). The frequency of HAT^R^ colonies formed in this step, therefore, provides a measure of *I-Sce*I-induced accNHEJ. (Like other accNHEJ reporter assays (10,17–20), this scheme involves the deletion of DNA between two *I-SceI* sites, but measures accNHEJ rather than mutNHEJ because the joined ends suffer no gain or loss of nucleotides.) Finally, the resulting HAT^R^ colonies can then be used to generate 6TG^R^ colonies as a measure of *I-Sce*I-induced mutNHEJ (Fig. 3Bc).

### *I-Sce*I-induced accNHEJ at the human *HPRT* locus

*HPRT*^+^ HT1080 cells were electroporated with pLB-puro and clones were selected in 6TG and puromycin (Fig. 3Ba). From a total of 20 million cells, 12 clones were obtained, consistent with the low un-induced targeting frequencies in HT1080 cells (47). One such clone (clone 5.2) was then electroporated with *I-Sce*I expression plasmid, or mock transfected, and only the former transfection generated HAT^R^ clones (not shown). Southern analyses of a pool of >200 HAT^R^ colonies (clone 5.2P), two HAT^R^ clones (clones 5.2.1 and 5.2.2; both sensitive to puromycin) and parental clone 5.2 confirmed the presence of the *I-Sce*I site in exon 6 (Supplementary Material, Fig. S2A and B). To estimate the frequency of accNHEJ, multiple lipofections of clone 5.2 with *I-Sce*I expression plasmid were carried out and the frequency of resulting HAT^R^ colonies was measured at 4.1% [standard deviation (SD): 1.15; *n* = 4]. Among these HAT^R^ colonies, 4.4% (SD: 0.59; *n* = 4) remained Puro^R^, presumably due to reintegration of the excised puromycin cassette at random genomic loci. These results support the use of clone 5.2, or equivalent clones, in assays for accNHEJ as outlined (Fig. 3Bb). Though highly specific, this assay detects only accNHEJ events between two DSBs, excluding the probably more frequent accNHEJ events involving only one DSB (see Discussion).

### *I-Sce*I-induced mutNHEJ at the human *HPRT* locus

**Figure 4.**
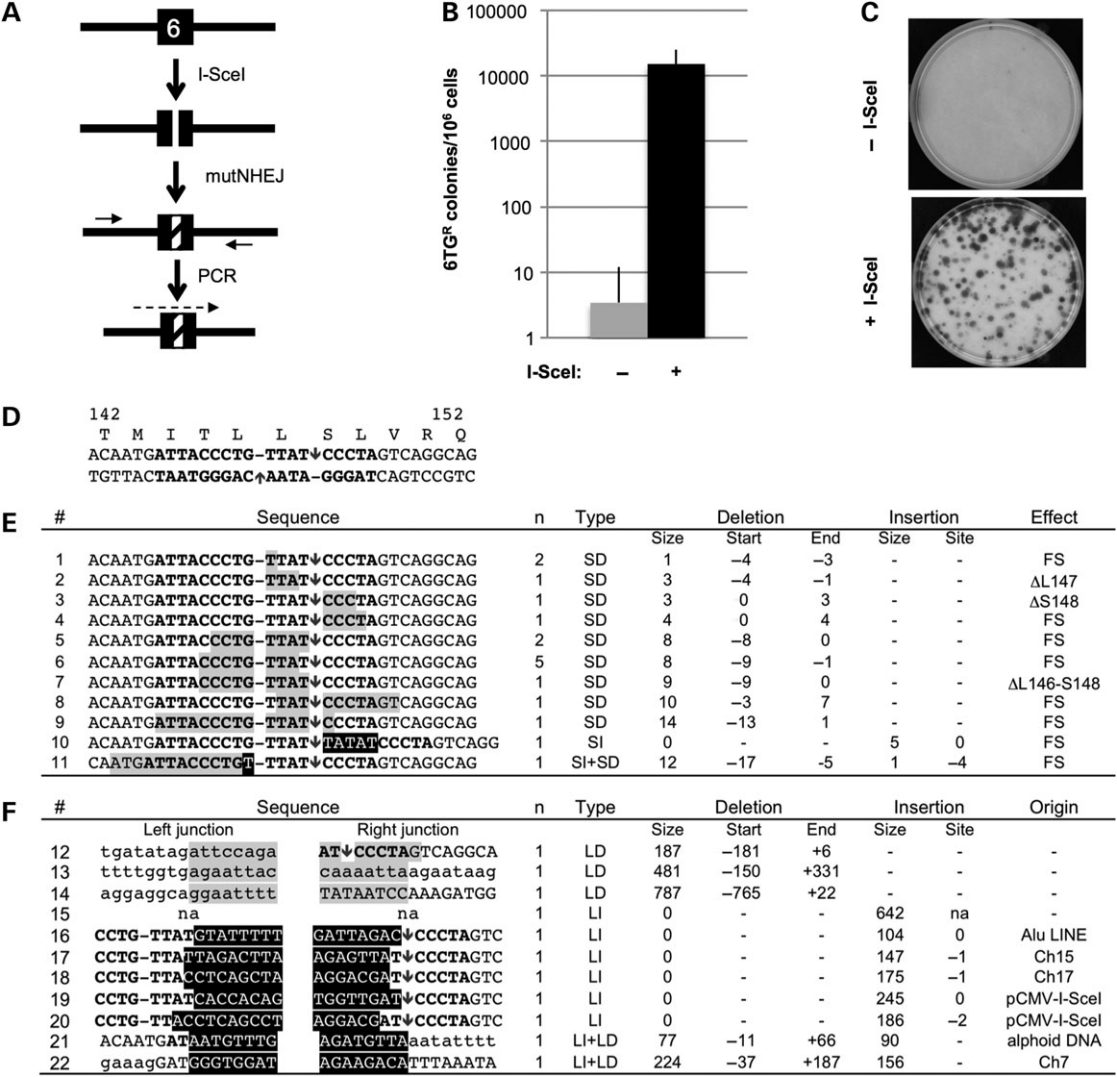
Assays for *I-Sce*I-induced mutNHEJ and DNA sequence analyses of resulting indels in *HPRT* exon 6. (**A**) *HPRT* exon 6 in clone 5.2.1 is cleaved by *I-Sce*I and repaired by mutNHEJ to generate 6TG^R^ clones carrying exon 6 indels (hatched); these are amplified with flanking primers (short arrows) and sequenced (dashed arrow). (**B**) Frequency of 6TG^R^ clones generated from clone 5.2.1 lipofected with *I-Sce*I expression plasmid or a vector control (mean and SD for six experiments are shown). Transfection was by lipofectamine 2000, but similar results were obtained with Fugene. (**C**) Example of 6TG^R^ colonies forming in the experiment in B. Petri dishes (10 cm diameter) were seeded with 10^5^ cells before selecting in 6TG. (**D**) Part of the exon 6 DNA sequence in clone 5.2.1 is shown aligned with encoded amino acids 142–152. The recognition site for *I-Sce*I is shown (bold) with positions of staggered nicks it makes (arrows) and un-cleaved phosphodiester bonds opposite each nick (hyphens). (**E**) Small indels. Coding strand DNA sequences of 11 indels identified in seventeen 6TG^R^ clones are shown with deleted and inserted residues highlighted in grey and black, respectively. The number of clones for each indel is shown (*n*). Positions of indels are summarized with residues numbered relative to the coding strand nick. Micro-homologies likely to have mediated deletion formation are underlined. Indel types: small and large deletions (SD, LD), or insertions (SI, LI). The predicted effects on protein coding are categorized as frame-shifts (FS) or in-frame deletions (Δ). (**F**) Large indels. Sequences of 11 junction regions are shown with DNA represented as in (B). Except for insertions, upper and lower case letters represent exon 6 and intronic residues, respectively. Type and position numbers of indels are summarized as in (B). The origin of the inserted DNA is indicated. na, no sequence available.

When transfected with an *I-Sce*I expression plasmid, clone 5.2.1 (*HP-I-RT^+^*) generated 6TG^R^ colonies, as expected for DSBR by mutNHEJ resulting in indels (Fig. 4A). In standard lipofection protocols, 6TG^R^ colonies were generated at frequencies of 1.5% (SD: 0.96, *n* = 8), three to four orders of magnitude higher than the frequency of spontaneous 6TG resistance (Fig. 4B and C). To characterize the expected indels, the exon 6 regions of 28 individual 6TG^R^ clones were amplified and sequenced (Fig. 4A and D–F). The majority (17/28) had small indels involving <14 nt, all but two of which were simple deletions. The rest involved larger deletions (3/28), insertions (6/28) or both (2/28). These results support the use of the *I-Sce*I-sensitive *HPRT^+^* allele in clone 5.2.1 (or equivalent clones), in assays to detect DSB-inducible mutNHEJ.

### *I-Sce*I-induced correction of the human *HPRT* gene

**Figure 5.**
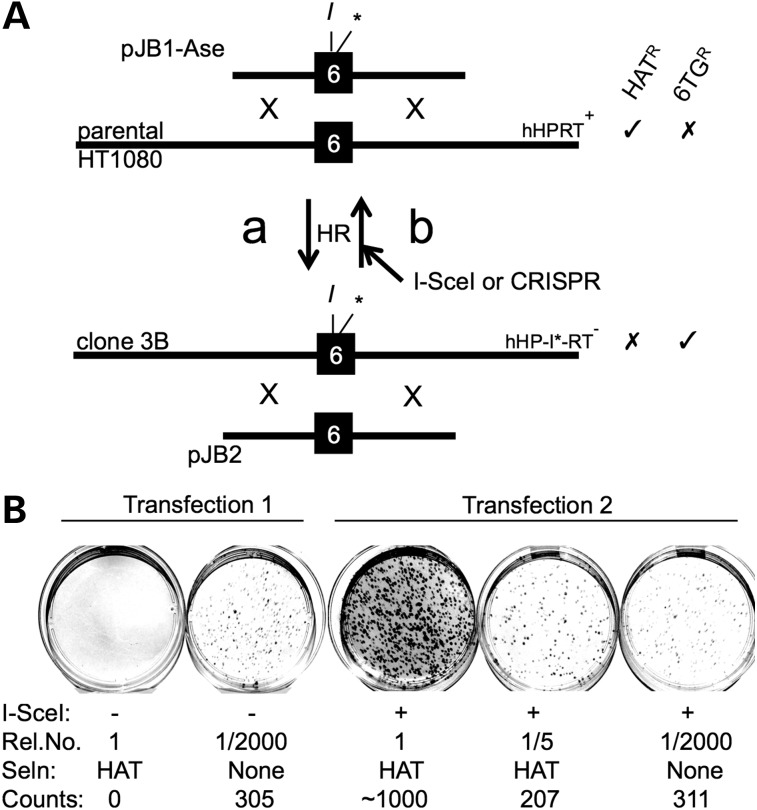
Generating and using *I-Sce*I-sensitive *hHPRT^+^* alleles for gc/HR assays. (**A**) DNA is represented as in Figure 3. (**a**) HR between a wt *HPRT* allele and the targeting construct pJB1-*Ase*I was used to introduce an *I-Sce*I site and adjacent stop codon (*) into exon 6. The resulting cells (e.g. clone 3B) were selected in 6TG. (**b**) Basis of assays for gene correction by HR (gc/HR). Clone 3B cells are transfected with a repair template (pJB2) and nuclease expression plasmid(s). HR with transfected pJB2 regenerates a functional exon 6 and the frequency of resulting HAT^R^ colonies measures gc/HR. (**B**) Example of one experiment to estimate gc/HR frequencies. Clone 3B cells were co-transfected with pJB2 and either vector DNA (Transfection 1) or *I-Sce*I expression plasmid (Transfection 2), and the indicated relative numbers (Rel. No.) of cells were placed in petri dishes to select in HAT for gene correction events or to determine plating efficiencies (no selection). Colonies appearing after ∼10 days were counted and used to estimate gc/HR frequencies of <0.00016% (Transfection 1) and 0.17% (Transfection 2).

As a model for nuclease-induced gene correction by HR (gc/HR), we used a GT plasmid (pJB1-Ase) to generate clones with an inactive *HPRT* allele (HP-I*-RT^−^) in which the silent *I-Sce*I site in exon 6 has an adjacent stop codon and *Ase*I site (Figs 3A and 5Aa; Supplementary Material, Fig. S2C). Ten million *HPRT^+^* cells electroporated with pJB1-Ase generated five 6TG^R^ colonies, four of which (clones 3A, 3B, 10B1 and 10B3) had *Ase*I fragments indicative of the desired modification (Supplementary Material, Fig. S2C). Southern analyses confirmed the presence of an *I-Sce*I site in exon 6 in each of three clones tested (3B, 10B1 and 10B3; Supplementary Material, Fig. S2A). Clone 3B was then used for gc/HR assays in which a repair construct (pJB2) was co-lipofected with an *I-Sce*I expression plasmid (or vector control), followed by selection in HAT (Fig. 5Ab). After normalizing for plating efficiencies, the frequency of HAT^R^ colonies, and therefore gc/HR, was found to be 0.16% (SD: 0.07; *n* = 6). Representative results for one experiment are shown in Figure 5B. Further experiments (not shown) confirmed that gc/HR requires co-transfected repair template and that clones 3A and 10B1 behaved similarly to clone 3B. These results support the use of the *I-Sce*I-sensitive *HPRT^−^* allele in clone 3B (or equivalent clones) in assays to detect DSB-inducible gene correction and show that this is ∼10-fold less efficient than repair of the same DSB by mutNHEJ.

### Cell cycle-specific control of *I-Sce*I-induced DSBR

**Figure 6.**
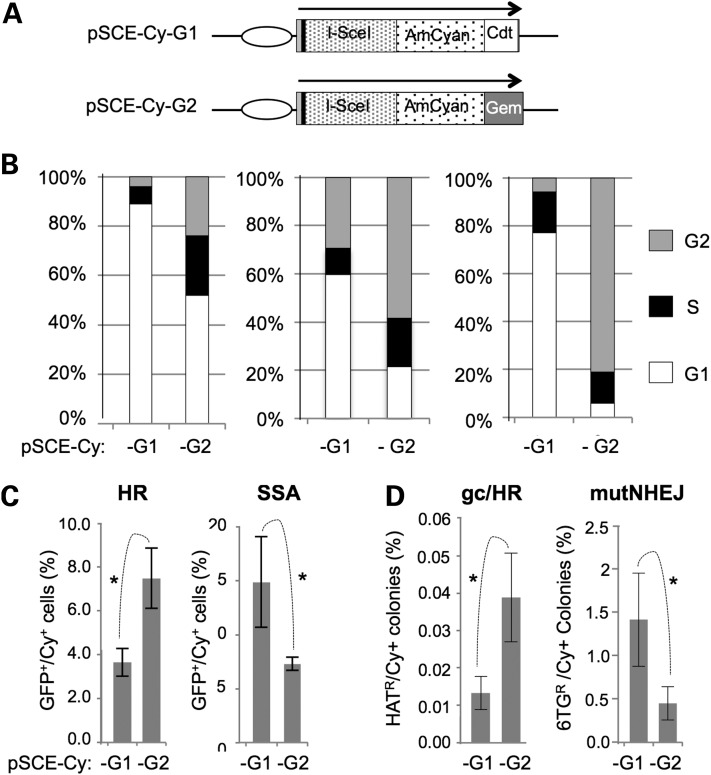
Development and use of cell cycle-specific *I-Sce*I. (**A**) Schematic representation of *I-Sce*I expression plasmids. Ellipse, cytomegaloviral promoter; black bar, haemagglutinin tag. (**B**) Cell cycle profiles of cells lipofected with the indicated plasmids. Cells were HT1080-WT (left), HT1080-AS5 (Supplementary Material, Table S1; centre) and HeLa (right). (**C**) Differential effects of cell cycle-restricted *I-Sce*I on reporters for HR and SSA. The indicated plasmids were transfected into cells (HT-DR-GFP-8 or HT-SSA-GFP; Supplementary Material, Table S1) carrying GFP-based reporters for a HR or SSA. The percentage of AmCyan^+^ cells that were GFP^+^ is shown. (**D**) Differential effects of cell cycle-restricted *I-Sce*I on mutNHEJ and gene correction at the *HPRT* locus. Assays based on clone 5.2.1 (mutNHEJ) or clone 3B (gene correction) were used with the indicated *I-Sce*I expression plasmids. Results were normalized to the amount of AmCyan expression. Mean and SDs for three or more assays are presented with significant differences marked (*P < 0.05; Student's *t*-test).

Cell cycle studies typically rely on synchronization methods that use drugs or serum starvation to arrest the cycle or physical separation by centrifugal elutriation or flow cytometric cell sorting. Problems with these methods include the induction of cell stress responses, variable sensitivities to serum starvation and the need for expensive equipment with experienced operators (48). We reasoned that, in the context of DSBR, the expression of nucleases in a cell cycle-restricted fashion might provide a simple alternative to such approaches. We, therefore, used Fucci technology (49) to develop plasmids encoding fluorescent *I-Sce*I derivatives that accumulate preferentially in G1/S (pSce-Cy-G1) or S/G2 (pSce-Cy-G2). These encode *I-Sce*I fused to AmCyan fluorescent protein (Cy) with C-terminal peptides derived, respectively, from Cdt1 and Geminin (Fig. 6A; Supplementary Material, Table S2). Analyses of DNA content in transiently transfected AmCyan-positive (AmCyan^+^) cells confirmed that pSce-Cy-G1- and pSce-Cy-G2-transfected cells were enriched in G1/S and S/G2, respectively (Fig. 6B). To investigate whether the constructs have differential effects on previously established DSBR assays, they were transfected into cells stably transfected with the reporters DR-GFP (13) and SA-GFP (16) for measuring, respectively, *I-Sce*I-inducible intrachromosomal HR and SSA (Fig. 6C). The DR-GFP signal was ∼2-fold greater in cells expressing pSce-Cy-G2 than in cells expressing pSce-Cy-G1, consistent with the known restriction of HR to S/G2. Conversely, the SA-GFP signal was ∼2-fold greater in cells expressing pSce-Cy-G1 than in cells expressing pSce-Cy-G2, consistent with studies suggesting that SSA does not occur in G2 (50) and that resection can occur in G1 (51). These results suggested that our Fucci *I-Sce*I constructs are able to introduce DSBs in restricted phases of the cell cycle, as intended. To assess how the Fucci *I-Sce*I constructs affect mutNHEJ and gc/HR at the *hHPRT* locus, we then transfected them into clones 5.2.1 (*HP-I-RT^+^*) and 3B (HP-I*-RT^−^), respectively (Fig. 6D). Similar to the DR-GFP assays, pSce-Cy-G2 induced gc/HR with ∼3-fold greater efficiency than pSce-Cy-G1. Conversely, pSce-Cy-G2 induced mutNHEJ ∼3-fold less efficiently than pSce-Cy-G1 suggesting that, although NHEJ operates throughout the cell cycle, mutNHEJ occurs preferentially in G1/S in HT1080 cells. Together, these observations support the use of our modified *I-Sce*I expression plasmids for studying the influence of cell cycle on any DSB-inducible cell response.

### Comparison of Cas9- and *I-Sce*I-induced DSBR at *HPRT* exon 6

**Figure 7.**
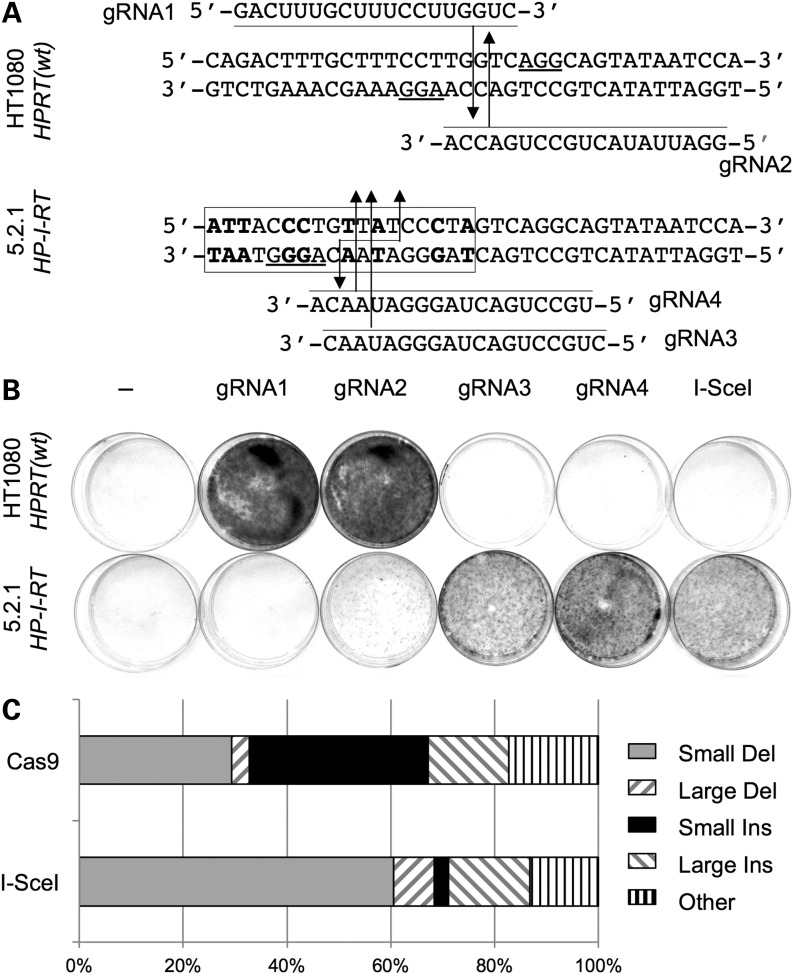
Cas9-induced *HPRT* mutagenesis. (**A**) Design of exon 6-targeted gRNAs. Part of the exon 6 sequence in WT cells or clone 5.2.1 is shown. The *I-Sce*I site is boxed with non-WT residues shown in bold and the cleavage site indicated (staggered arrows). Target-specific regions of gRNAs are shown, aligned with their target. For each gRNA, the predicted Cas9 cleavage site is indicated by an arrow, 3 nt upstream of the protospacer-adjacent motif (underlined). (**B**) Stained petri dishes after selection of transfected HT1080 or clone 5.2.1 cells (10^6^ cells per dish) in 6TG. Cells were lipofected with DNA encoding no nuclease (column 1), Cas9 and the indicated gRNA (columns 2–5), or *I-Sce*I (column 6). Cells from the same transfection shown in (B) were plated at lower dilutions and selected in 6TG and used to determine frequencies (Materials and Methods; Table 3, Experiment 1). (**C**) Relative proportions of different classes of *I-Sce*I- or Cas9-induced mutations (data from Fig. 4D and E; Supplementary Material, Fig. S3).

To develop *HPRT*-based DSBR assays that do not rely on introducing an *I-Sce*I site into the genome, and to compare *I-Sce*I and CRISPR-induced DSBR pathways, we designed four exon 6-specific guide RNAs for the Cas9 nuclease of *Streptococcus pyogenes*: two (gRNA-1 and -2) targeting wild-type *HPRT* alleles and two (gRNA-3 and -4) targeting *I-Sce*I-sensitive *HPRT* alleles (Fig. 7A). To test for Cas9-induced mutNHEJ, wild-type cells or *I-Sce*I-sensitive *HPRT^+^* cells (clone 5.2.1) were co-transfected with expression plasmids for Cas9 and each of the four gRNAs, and 6TG^R^ colonies were selected. For a positive control, the *I-Sce*I expression plasmid was transfected in parallel. The results (Fig. 7B, Table 2) clearly showed the expected gRNA targeting preferences. Thus, high frequencies of 6TG^R^ colonies (up to 3.6-fold higher those induced by *I-Sce*I in clone 5.2.1) were induced by gRNA-1 and -2 in wild-type cells, and by gRNA-3 and -4 in clone 5.2.1 cells. Conversely, no 6TG^R^ colonies were generated in wild-type cells by gRNA-1 or in clone 5.2.1 by gRNA-3 or -4. These results indicate stringent allele-specific exon 6 mutagenesis directed by gRNAs 1, 3 and 4. Mutagenesis directed by gRNA-2, though highly efficient in clone 5.2.1, was also appreciable in parental cells. This most likely reflects the fact that gRNA-2 has only one mismatch to the wild-type allele and so can direct Cas9-mediated cleavage, albeit less efficiently than at the *I-Sce*I-sensitive allele.

**Table 2.** Relative frequencies of *HPRT* mutagenesis by Cas9 and *I-Sce*I^a^

Expt.	Host cells	*HPRT* genotype	Nuclease					
			None	gRNA1/Cas9	gRNA2/Cas9	gRNA3/Cas9	gRNA4/Cas9	*I-Sce*I
1	WT	*HPRT^+^*	UD	1.5	1.7	UD	UD	UD
1	5.2.1	*HP-I-RT^+^*	UD	UD	0.05	1	3.6	1
2	5.2.1	*HP-I-RT^+^*	nt	UD	nt	1.3	nt	1
3	5.2.1	*HP-I-RT^+^*	nt	nt	nt	0.5	1.4	1
4	WT	*HPRT^+^*	UD	2.6	nt	UD	UD	UD
4	5.2.1	*HP-I-RT^+^*	UD	UD	UD	1.6	2.9	1

UD, undetectable (<10^−5^); nt, not tested.

^a^Figures show the frequencies of 6TG^R^ colonies generated by the indicated nucleases relative to frequencies generated by *I-Sce*I.

We sequenced the exon 6 region in a range of 6TG^R^ clones generated in the experiment of Figure 7B and in similar experiments (Supplementary Material, Fig. S3). For both Cas9 and *I-Sce*I, indels occurred at the expected sites. We classified these indels, and those of Figure 4, as small or large deletions or insertions, or other more complex events. We noted a striking difference in the frequency of small insertion mutations generated by *I-Sce*I and Cas9 (Fig. 7C): small insertions represented the least abundant class of *I-Sce*I-generated mutations (1/38; 2.6%), but the most abundant class of Cas9-generated mutations (20/58; 34.5%).

Finally, we tested gRNA-1, -3 and -4 for their ability to support Cas9-induced gene correction in *I-Sce*I-sensitive *HPRT^−^* cells (clone 3B). As expected, gRNA-3 and -4, but not gRNA-1, supported Cas9-mediated gene correction (Table 3). Also as expected, detectable levels of correction induced by gRNA3/Cas9 depended, as for *I-Sce*I, on template co-transfection. Interestingly, Cas9-induced gene correction was ∼7-fold less efficient than *I-Sce*I-induced correction. This contrasted with Cas9-induced mutNHEJ that was on average 1.7-fold more efficient than *I-Sce*I-induced mutNHEJ (Table 2). Together with the differences in mutNHEJ mutation signatures (Fig. 7C), these results suggest that Cas9- and *I-Sce*I-generated DSBs are processed differently during DSB repair.

**Table 3.** Frequencies of gc/HR induced by different HPRT-targeted nucleases^a^

Nuclease	*I-Sce*I	*I-Sce*I	gRNA1/Cas9	gRNA3/Cas9	gRNA3/Cas9	gRNA4/Cas9
Template	−	+	+	−	+	+
Experiment 1	UD	0.093	UD	UD	0.013	nt
Experiment 2	nt	0.12	nt	nt	0.016	0.019

UD, undetectable; nt, not tested.

^a^Figures show % of colony forming units that became HAT^R^ after lipofection of clone 3B with the indicated nucleases with or without repair template (pJB2).

## Discussion

In this study, we have developed a system for measuring the repair of *I-Sce*I- or Cas9-induced DSBs by three different mechanisms, all at the same site in the endogenous *HPRT* gene. We have used these assays to estimate the absolute and relative frequencies of the different DSBR mechanisms, to discover that *I-Sce*I and Cas9 induce distinct mutNHEJ mutation signatures and mutNHEJ:GT ratios, and to validate novel *I-Sce*I derivatives for studying DSBR in different cell cycle phases. In addition, we have used an *I-Sce*I-sensitive *HPRT* minigene to compare the efficiencies of different DNA repair templates.

### Advantages and limitations of DSBR assays using the endogenous *HPRT* locus

A key attraction of the endogenous *HPRT* assay system is its ability to measure three DSBR pathways (GT, mutNHEJ and accNHEJ) at the same site. As outlined in the Introduction, the comparison of multiple DSBR pathways at a single locus is preferable to the comparison of single pathways at multiple loci. This is because the interactions between a reporter and its chromosomal environment, and any associated effects on DSBR, vary unpredictably between loci. Several studies have compared two DSBR pathways at a single locus but, to our knowledge, detection of three pathways at a single locus has been described only once, for a different combination of pathways (GT, mutNHEJ and SSA) using a TLR construct (26).

For mutNHEJ, the endogenous *HPRT* system has the advantage of being able to detect a full spectrum of indels. Thus, by cleaving an essential *HPRT* coding region, we detected single nucleotide indels and in-frame deletions of just a single codon (Fig. 4D). Furthermore, we detected indels of over 600 bp (Fig. 4F) closer in size the *HPRT* mutations commonly detected after ionizing irradiation (43). Most mutNHEJ assays use gain-of-function reporters whose reading frame must be restored so that, at best, only one-third of all possible indels can be detected. Furthermore, many large indels are expected to prevent reporter cassette activation, for example, by deleting into its promoter or coding sequences, or by the inserting exogenous sequences that impair expression. In a loss-of-function mutNHEJ assay based on the endogenous thymidine kinase gene, *I-Sce*I cleaved a non-coding region and so was unable to detect all indels (52).

Like other reporters, the endogenous *HPRT* system requires two *I-Sce*I sites to detect accNHEJ, but it is unusually selective because, as shown in our mutNHEJ assays, even the smallest indels are incompatible with HAT resistance. This contrasts with other reporter systems where small indels were compatible with reporter gene expression, so that DNA sequence analyses were required to distinguish accNHEJ from mutNHEJ (10,17–20). In common with most other accNHEJ reporters, however, our *HPRT*-based assay underestimates the true level of accNHEJ, partly because multiple cycles of accNHEJ and re-cleavage increase the chance that DSBR will ultimately occur by mutNHEJ. This issue was addressed in another study by use of an exogenous reporter in which accNHEJ of two mutant *I-Sce*I generates a site resistant to further cleavage (10), a feature that could readily be incorporated into the *HPRT* assay. Nevertheless, another cause for accNHEJ underestimation, lack of reporter activation by accNHEJ at only one of the two *I-Sce*I sites, remains to be addressed in any accNHEJ assay.

Assaying for GT at the cleaved *HPRT* locus is, like the mutNHEJ and accNHEJ assays, highly selective. *HPRT* can, therefore, report for these different DSBR mechanisms without the need for DNA sequencing of multiple clones, an advantage shared with TLRs. Although selection and scoring of drug-resistant colonies is less convenient than analyses of fluorescent reporters, the *HPRT* system does not require a multicolour flow cytometer and conveniently delivers clones ready for any further analyses that might be required. Furthermore, the *HPRT* assay is highly sensitive, easily capable of unambiguously detecting one event among a million or more cells.

The fact that *HPRT* is an endogenous housekeeping gene also has attractions. First, use of *HPRT* avoids the need to integrate an exogenous reporter into the genome and to demonstrate single copy integration. Indeed, measurements of Cas9-induced mutNHEJ can be carried out without any preparatory modifications of the host cells. Although *HPRT* modifications, such as the introduction of an *I-Sce*I site, are required for measuring accNHEJ and GT, the reagents and selection steps we have described should be applicable in most cell lines. Second, as outlined above, the fixed genomic location of *HPRT* is an advantage if one wishes to compare DSBR in different cell types. Similarly, the conservation of *HPRT* allows for well-controlled comparisons of DSBR between cells from different species. Although a fixed location precludes studies of chromosome position effects on DSBR, for this purpose, an *HPRT* minigene or bacterial artificial chromosome carrying the whole *HPRT* gene can be ectopically integrated, with the endogenous locus serving as a reference. Third, the endogenous *HPRT* system allows DSBR to be studied at a natural gene with features such as multiple introns and repeat sequences intact and unmodified by exogenous sequences that may affect DSBR.

### Relative frequencies of different DSBR pathways

Our estimated efficiencies of *I-Sce*I-induced mutNHEJ and GT the endogenous *HPRT* locus in HT1080 (human fibrosarcoma) cells were 1.5 and 0.16%, respectively, giving a mutNHEJ:GT ratio of ∼9:1. In a previous study, where different chromosome positions of the mutNHEJ and GT reporters were taken into account, the *I-Sce*I-induced mutNHEJ:GT ratio was estimated at random loci in HCA-2 (human colon cancer) cells to be 6:1 (23). In another study, where chromosomal position effects were avoided by use of a TLR, the average mutNHEJ:GT ratios were estimated at transcribed and untranscribed loci in HEK293 (human embryonic kidney) cells to be 4:1 and 25:1, respectively (26). For comparison with our estimate, these estimates must be increased 3-fold (because their mutNHEJ reporters detect only in-frame indels), to give ratios of 18:1, 12:1 and 75:1, respectively. Thus, despite the differences in variables such as cell type and DNA delivery method, our estimate of 9:1 is in good agreement with the value of 12:1 corresponding to a pool of transcribed loci derived from the study of Kuhar *et al*. This suggests that the endogenous *HPRT* locus is representative of an average transcribed locus, when measuring DSBR.

Our estimated efficiencies of *I-Sce*I-induced accNHEJ and mutNHEJ at the *HPRT* locus in HT1080 cells were 4.1 and 1.5%, respectively, giving an accNHEJ:mutNHEJ ratio of ∼3:1. As discussed above, although our accNHEJ and mutNHEJ assays are particularly specific, they are also likely, in common with other assays, to underestimate this ratio, partly because of re-cleavage of accurately repaired DSBs. Thus, our estimate is a robust only as a minimum estimate; true values require further investigation and are likely to be much higher. Perhaps the best estimate to date (∼50:1) used an accNHEJ substrate that cannot be re-cleaved by *I-Sce*I after accNHEJ (10). Further estimates are required that not only compensate for re-cleavage by nuclease but also detect accNHEJ at a single DSB.

### Differences between *I-Sce*I- and Cas9-induced DSBR

It was surprising that, compared with *I-Sce*I, Cas9 induced higher frequencies of mutNHEJ and lower frequencies of GT, even though both nucleases targeted the same regions in *HPRT* exon 6. It was also striking that Cas9, unlike *I-Sce*I, had a marked propensity to generate small (mostly 1 nt) insertions. A high proportion of single nucleotide insertions among Cas9-induced indels has been noted previously (53,54), but its significance is unclear. It is possible that these differences between Cas9- and *I-Sce*I-induced DSBR reflect the different DNA ends they generate. Thus, the recessed 5′ ends generated by *I-Sce*I may be more prone than blunt Cas9-generated ends to undergo further 5′ end-resection, favouring RAD51 filament formation and GT at the expense of mutNHEJ. This interpretation is also consistent with recent data showing that a customized nuclease that generates protruding 5′ ends stimulated GT less efficiently than cleavage of the same reporter by *I-Sce*I (26). The high proportion of single base insertions among Cas9-induced indels is accompanied by a decrease in the proportion of small deletions (Fig. 7C). This could be explained if blunt ends are less susceptible to nuclease digestion than 3′-overhangs, and so more susceptible to nucleotide addition. Consistent with this, chromosomal translocations induced by wild-type Cas9 involve deletions less frequently than natural translocations or translocations induced by paired Cas9 nickases (55). Further investigations of how DSBR pathways are affected by different nuclease and DSB end-structures will be valuable for elucidating natural mutagenesis and optimizing genome engineering methods.

The ease with which CRISPRs can be developed will facilitate further analyses of DSBR at various regions in the *HPRT* locus. The ability to place a silent *I-Sce*I site in exon 6 will nevertheless remain a valuable option for studies of DSBR using *HPRT* as a reporter. For example, it will allow comparisons of DSBR at the *HPRT* locus in different species using a single defined nuclease, or studies of the influence of effector proteins, targeted to the *HPRT* locus by fusion to a nuclease-inactive Cas9, on *I-Sce*I-induced DSBR.

### Cell cycle regulated *I-Sce*I

Fusion of Geminin- or Cdt1-derived peptides to AmCyan-tagged *I-Sce*I allowed us to induce DSBs preferentially in S/G2 and G1/S, respectively. This approach offers a relatively simple and non-invasive way to study how any DSB-induced cellular response varies with cell cycle and adds to previous modifications of *I-Sce*I designed to extend its versatility as a tool in the study of DSBR (24,56,57). So far, we have used our approach to demonstrate cell cycle regulation of intrachromosomal HR, SSA, gc/HR and mutNHEJ, but it will be interesting to extend this to other DSBR pathways, including accNHEJ. It should be noted that DSBR assays preferentially activated by a phase-specific *I-Sce*I, while indicative of phase-specific DSBR, do not always indicate that DSBR occurs in that phase. In cells with defects in NHEJ and cell cycle checkpoints, for example, DNA damage inflicted in one cell cycle phase may be repaired in a subsequent phase (58).

A similar approach to that described here may be applicable to CRISPRs with potential implications for genome engineering. For example, the use of S/G2-restricted nucleases may provide a way to enhance the proportion of any desired HR-mediated genomic modifications at the expense of unwanted NHEJ-mediated events. In our study, AmCyan^+^ cells were enriched in G1/S or G2/S phases, but the absolute numbers of AmCyan^+^ phase-enriched cells were limited. It will, therefore, be important to optimize levels of *I-Sce*I-fusion protein expression if these reagents are to be fully exploited.

### Use of a *HPRT* minigene reporter

Although our initial experiments involved an ectopic mouse HP-I*-RT minigene and were not used to measure absolute frequencies of gene correction, they did generate several interesting results concerning the mechanisms of GT efficiencies using different templates. First, we measured similar frequencies of DSB-induced gene correction for sense and antisense single-stranded oligonucleotides (ssOs). This could not have been predicted from a similar result for un-induced ssO-directed modifications (59), which are thought to involve ssO annealing at replication forks (60,61) rather than at resected DSBs, as is envisaged for nuclease-induced DSBR (62). Our results, therefore, suggest that resection and oligonucleotide annealing on each side of the DSB are equally efficient. Second, the higher efficiency of ssO- relative to dsO-templated correction suggests that ssO annealing at resected DSB ends is preferred over invasion of the dsO by the resected ends, as is envisaged for long double-stranded templates (see 63). Third, the >20-fold increase in GT on increasing the ds template length from 60 to ∼1700 bp is of note. Un-induced GT efficiencies increase exponentially with template length (64), probably because the chance that a random chromosome DSB occurs within the homology region increases with template length. For nuclease-induced GT, however, this explanation does not apply. It may be, therefore, that increasing homology favours homology searching and/or strand invasion, or that long double-stranded templates are less susceptible to degradation than double-stranded oligonucleotides. Fourth, the greater effectiveness of wild-type templates compared with equivalent templates with *I-Sce*I sites was observed for both double- and single-stranded templates. This suggests that re-cleavage of the repaired chromosome by *I-Sce*I is more of a barrier to gene correction than cleavage by *I-Sce*I of double-stranded templates prior to HR. Finally, the greater efficiency of GT with long double-stranded templates than with oligonucleotides contrasts with other studies where ssOs supported nuclease-induced gene modifications with similar or greater efficiencies than plasmid templates (46,65,66). The reason for this difference is unclear, but it may reflect differences in optimal experimental conditions required for different loci and cell types. For example, although our oligonucleotide concentrations were similar to those used by others (46), they were not necessarily optimal. Indeed, increasing oligonucleotide concentrations enhanced correction frequencies up to 4-fold (Supplementary Material, Fig. S3).

In summary, we have demonstrated the versatility of using *HPRT* to investigate nuclease-induced DSBR at an endogenous locus. Although nucleases have previously been used to induce DSBR at endogenous selectable genes (52,67–71), our study uniquely combines the analysis of three different types of DSBR at an endogenous locus, the use of *I-Sce*I to cleave a functional exon (allowing a fuller range of DSBR events to be selected) and the comparison of *I-Sce*I- and Cas9-induced DSBR. The use of an exogenous *HPRT* minigene to study gene correction and of modified *I-Sce*I to study cell cycle-specific DSBR adds further novelty and utility to this study. These systems will be applicable in a wide range of investigations into DSBR.

## Materials and Methods

### Cell culture and transfection

HT1080 fibrosarcoma cells and derivatives and HeLa cells were grown as previously described (72). Supplementary Material, Table S1 summarizes key properties of various HT1080 derivatives used. For electroporations, a Gene Pulser (BioRad) was used as previously described (72). For lipofections, lipofectamine 2000 (LF; Life Technologies) or Fugene 6 (Promega) was used according to the manufacturers’ instructions, in multiwall well formats with, per cm^2^, 0.5–1 million cells, a total of 0.4 µg (LF) or 0.6 µg (Fugene) DNA and 1 µl (LF) or 1.8 µl (Fugene) cationic lipid reagent. Under these conditions, 70–90% of HT1080 cells lipofected with a green fluorescent protein (GFP) expression plasmid became GFP^+^ 24–48 h post-lipofection. For nucleofection, an Amaxa Nucleofector I (Lonza) was used according to the manufacturer's recommendations (program L005, transfection solution T).

### Drug selections and colony counts

To select for stably transfected/modified clones or populations, the following drug selections were used: hygromycin B (Invitrogen; 100 µg/ml), zeocin (Invivogen 200 µg/ml), puromycin (Invivogen; 0.4 µg/ml), HAT supplement (Life technologies) and 6TG (Sigma; 15 µg/ml). After transfection, but prior to selecting in 6TG, cells were maintained without selection for 5 days, passaging as necessary. Other selective agents were added to transfected cells 48 h post-transfection. Because 6TG and HAT selection are initiated 48 h or more after transfection, by which time most DSB is complete (14), any influence of HAT or 6TG on DSBR is minimized. To determine frequencies of HAT^R^ or 6TG^R^ cells, colonies were selected in 9 cm diameter dishes over a range of cell dilutions. To determine plating efficiencies, a high dilution was plated without selection. Colonies were stained with crystal violet as described (72). Dilutions generating appreciable but limited numbers of HAT^R^ or 6TG^R^ colonies (e.g. 50–300) were chosen for colony counting. To allow for plating efficiency variations, the number of selected colonies in a given dish was divided by the number of colony forming units in that dish, as calculated from the number of colonies that formed without selection at a known dilution of the same culture.

### DNA manipulations and plasmids

Key features of the plasmids used in this study are summarized in Supplementary Material, Table S2. Standard recombinant DNA methods were used for plasmid construction. Correct assembly was confirmed by restriction enzyme mapping and/or DNA sequencing. Details of construction and/or plasmid maps are available on request. The following kind gifts were made—*mHPRT* minigene vector pBT/PGK-HPRT (73): David Melton (Edinburgh University); *I-Sce*I expression plasmid pCMV3xnls-*I-Sce*I (74) and reporter constructs DR-GFP (13) and SA-GFP (16): Maria Jasin (Memorial Sloan-Kettering Cancer Center) and expression constructs for gRNA (Addgene 41824) and Cas9 (Addgene 41815): George Church (Harvard Medical School, via Addgene). Cloning of gRNA into 41824 was as described (75).

### Oligonucleotides, polymerase chain reaction and DNA sequencing

Oligonucleotides used as mHP-I*-RT repair templates are described in Supplementary Material, Table S3. To characterize indels, *hHPRT* exon 6 regions were amplified as follows. A 1433 bp region was amplified with primers P1 (5′-AGGGAACCCTTCTGTGTGTG-3′) and P2 (5′-GGACAATTCCCTATGCCTCA-3′) under the following conditions: 120 min at 94°C, then 30 cycles of 10 min at 94°C, 30 min at 60°C, 80 min at 72°C, then 120 min at 72°C. Alternatively, a 507 bp region was amplified with primers P3 (5′-GGCATTCTTACTGCTTGCTG-3′) and P4 (5′-TCTGCCATGCTATTCAGGAC-3′) under same conditions except with 30 min at 72°C instead of 80 min. Polymerase chain reaction products were purified and sequenced with primer P3 or P4.

### Southern analyses

Use of genomic DNA (preparation, digestion, electrophoresis and transfer to nylon membranes) and probes (^32^P-labelling and hybridizations to membranes) were essentially as described previously (72). The *hHPRT* probe was a 654 bp *Xba*I–*Bam*H1 fragment of *hHPRT* genomic DNA including exon 5 and part of intron 5. The puromycin probe was a 1.2 kb *Xba*I fragment from pBL-Puro/R (76).

### Flow cytometry

Measurements of GFP fluorescence alone were made in a Facscalibur (Beckton Dickinson; 488 nm laser). Measurements of AmCyan fluorescence in combination with propidium iodide (PI) or GFP fluorescence were made in an LSRII flow cytometer (Becton Dickinson; 405 and 488 nm lasers). To determine cell cycle profiles, cells were washed in phosphate buffered saline A (PBSA) and fixed by drop-wise addition of 500 µl cold (−20°C) 70% (v/v) ethanol, with vortexing, and stored at −20°C. Fixed cells were washed in PBSA at 40°C and incubated in RNaseA (10 µl; 1 mg/ml) for 30 min on ice, then resuspended in PI (200 µl, 50 µg/ml). For transfected cells, PI fluorescence was measured in populations gated for AmCyan fluorescence.

### *mHPRT*-based gene correction assays

Clone 2.1 cells were grown with (*I-Sce*I off) or without (*I-Sce*I on) tetracycline (Sigma; 1 µg/ml) for 48 h prior to nucleofection. Unless stated otherwise, each nucleofection used 250 000 cells with 5 µg of oligonucleotide or plasmid template. After nucleofection, cells were passaged for 5 days, with or without tetracycline. The entire population was then selected in HAT for 10–14 days and the resulting HAT^R^ colonies were counted.

### hHPRT-based DSBR assays

To measure *I-Sce*I-induced accNHEJ, clone 5.2 cells were lipofected with pCMV3xnls-*I-Sce*I and the frequency of resulting HAT^R^ colonies was determined. To measure I-SceI-induced mutNHEJ, clone 5.2.1 was lipofected with pCMV3xnls-*I-Sce*I and the frequency of resulting 6TG^R^ colonies was determined. The same protocol was used to measure CRISPR-induced mutNHEJ in clone 5.2.1 cell or wt HT1080 cells, except that equal weights of gRNA- and Cas9-expression plasmids were co-transfected. To measure *I-Sce*I- or CRISPR-induced gc/HR, clone 3B was lipofected with equal amounts of pCMV3xnls-*I-Sce*I and repair template (pJB2) or equal amounts of plasmids encoding gRNA, Cas9 and *I-Sce*I. The frequency of resulting HAT^R^ colonies was then determined.

### Assays for *I-Sce*I-AmCyan-induced DSBR

For mutNHEJ and gc/HR assays induced by *I-Sce*I-AmCyan fusions, part of the transfected culture was analysed 48 h post-lipofection to determine the proportion of AmCyan^+^ cells (typically <1%). Frequencies of mutNHEJ and gc/HR were then divided by their corresponding fraction of AmCyan^+^ cells. Cell lines HT-DRGFP-8 and HT-SAGFP-6 lipofected with *I-Sce*I-AmCyan expression plasmids were analysed flow cytometrically 48 h post-lipofection. After gating on 50 000–100 000 AmCyan-expressing cells, the proportion that also expressed GFP was measured.

## Supplementary Material

Supplementary Material is available at *HMG* online.

## Funding

This work was supported in part by the Biotechnology and Biological Research Council (BB/H003371/1 to A.C.G.P.), the Medical Research Council (MC_PC_12003 to T.C.H.), Cancer Research UK (C5255/A15935 to S.A.) and University of Oxford (Clarendon Scholarship to S.A.). Funding to pay the Open Access publication charges for this article was provided by the Research Councils UK open access fund.

## Supplementary Material

Supplementary Data
