# Supplementary material for: Use of the HPRT gene to study nuclease-induced DNA double-strand break repair
Source: Hum Mol Genet. 2015 Sep 30;24(24):7097–110. doi: 10.1093/hmg/ddv409 (PMC4654060; doi:10.1093/hmg/ddv409)
Supplement: Supplementary Data [file supp_24_24_7097__index.html]

Use of the HPRT gene to study nuclease-induced DNA double strand break repair — Use of the HPRT gene to study nuclease-induced DNA double-strand break repair — Use of the HPRT gene to study nuclease-induced DNA double-strand break repair — Supplementary Data 

# Use of the *HPRT* gene to study nuclease-induced DNA double-strand break repair

## Supplementary Data

Supplementary Data

- Supplementary Data - Pdf file
