# Supplementary material for: Use of the HPRT gene to study nuclease-induced DNA double-strand break repair
Source: Hum Mol Genet. 2015 Sep 30;24(24):7097–110. doi: 10.1093/hmg/ddv409 (PMC4654060; doi:10.1093/hmg/ddv409)
Supplement: Supplementary Data [file supp_ddv409_ddv409supp.pdf]

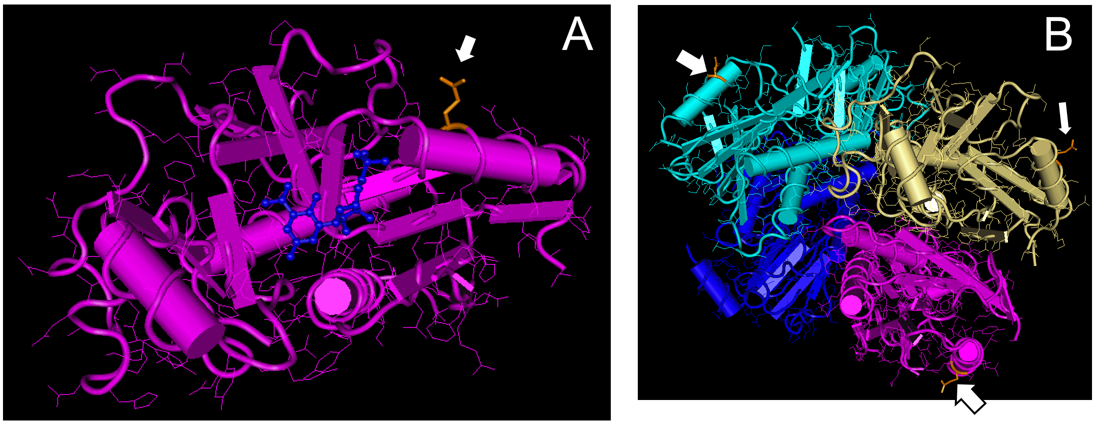

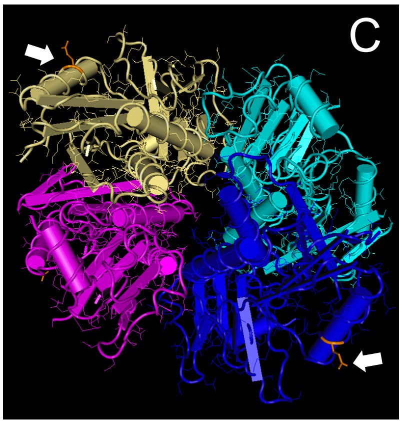

D

Human	LIVEDIIDTGKTMQTLLSLVRQYNPKM	HPRT_HUMAN
Grass mouse	LIVEDIIDTGKTMQTLLSLVKQYNPKM	Q9JK76_AKOCU
Mouse	LIVEDIIDTGKTMQTLLSLVKQYSPKM	HPRT_MOUSE
Pig	LIVEDIIDTGKTMQTLLSLVKQHNPKM	Q9XSI4_PIG
Dog	LIVEDIIDTGKTMQTLLSLVKEHNPKM	J9NVT2_CANFA
Hamster	LIVEDIIDTGKTMQTLLSLVKRYNLKM	HPRT_CRIGR
*Sheep	LIVEDIIDTGKTMQALLSLVRRHNPKM	W5PJ63_SHEEP
Cattle	LIVEDIIDTGKTMQTLLALVKKHKPKM	Q1JP91_BOVIN
Chicken	LIVEDIIDTGKTMKTLLSLLKQYNPKM	HPRT_CHICK
Salmon	LIVEDIIDTGKTMKTLLQLLKQYNPKM	B5DFU2_SALSA
Cod	LIVEDIIDTGKTMMTLLQLLKQYKPKM	G8DZS4_GADMO
Platypus	LIVEDIIDTGKTMKTLLSLVKQYNPKM	O97876_ORNAN
Frog	LIVEDIIDTGKTMKTLLAMLKKYNPKM	Q6DDP6_XENLA
Zebrafish	LIVEDIVDTGKTMKALLDHVEAFKPKM	AAO60356.1
*Owl limpet	LIVEDIIDTGKTMIKLLEILQKYNPKS	V4ATW6_LOTGI
Potato rot	LLVEDIIDTGKTMAKLVPMLMEYEPES	W2IHT2_PHYPR
Streptococci	LFVEDIIDTGKTLKSLCELFREKKAAS	X6PYR0_9STRE
Plasmidium	LIVEDIIDTGKTLVKFCEYLKKFEIKT	Q03925_PLAFA

Figure S1. Three dimensional location and evolutionary variations of hHPRT Q145. Screen shots are shown of structures analysed by Cn3D software using published data for the human HPRT monomer (**A)** (ref 1) and homotetramer (**B,C**) (ref 2). Residue Q145 is highlighted in orange and arrowed where visible. For the monomer, bound substrate is highlighted in blue. The external location is consistent with tolerance of the Q145I mutation. **D.** Comparison amino-acid sequences of proteins from various organisms identified on the basis of the highly conserved HPRT motif LIVEDIIDTTGKT. Human Q145 and its equivalent residues in other organisms are boxed. Differences to the human sequence are in red. K, Q, and Q are all seen at the Q145 position in known HPRT proteins. Isoleucine is seen at the Q145 position in an Owl Limpet protein that is likely to be HPRT. Sequences were identified by FASTA searches and are from known HPRT proteins or unknown proteins (*), as indicated. UniProtKB identifiers are indicated to the right of the sequence.

1. Naesens, L. *et al.* Role of human hypoxanthine guanine phosphoribosyltransferase in activation of the antiviral agent T-705 (favipiravir). *Mol. Pharmacol.* **84,** 615–29 (2013).
2. Keough, D. T., Brereton, I. M., de Jersey, J. & Guddat, L. W. The crystal structure of free human hypoxanthine-guanine phosphoribosyltransferase reveals extensive conformational plasticity throughout the catalytic cycle. *J. Mol. Biol.* **351,** 170–81 (2005).

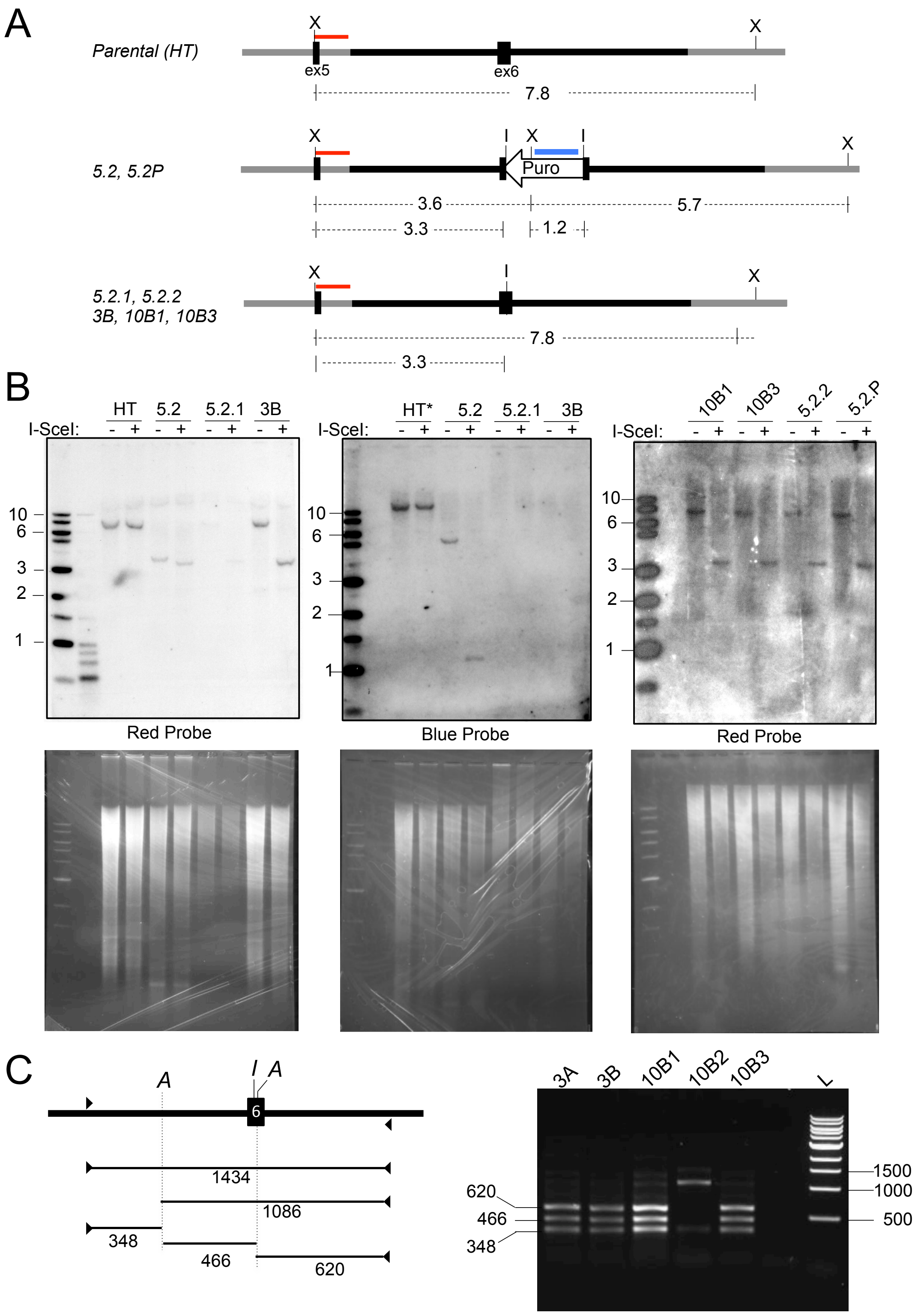

Figure S2 Molecular analyses of *hHPRT* loci. Legend on following page.

Figure S2 Molecular analyses of *hHPRT* loci. A. Scale maps of exon 6 regions in HT1080 cells and indicated derivatives. Exons 5 and 6 (black boxes) are indicated. Thick black and grey lines represent regions that, respectively, are or are not part of the targeting constructs described in Fig 2. The puromycin resistance cassette (Puro) is shown as a white arrow. Sites for XbaI (X) and *I-SceI* (I) are shown and fragments they generate are indicated (with sizes in kb) by dashed lines. Target- and construct-specific probes (red and blue bars, respectively) are shown aligned to their homologous regions. **B.** *Top*. Southern blots of gDNA of the indicated clones or pools of clones, digested with XbaI (with or without *I-SceI*) and probed with the indicated probes. Size markers (sizes shown in kb) were included in the first lane of each blot. The left-hand blot was stripped and re-probed to generate the central panel. Lanes 'HT' contain gDNA from HT-HPRT:hyg cells (Table S1) in which exon 2 is disrupted by a hygromycin-resistance cassette. Because the Hygro and Puro cassettes are partly homologous, the target-specific probe detects a 20 kb XbaI fragment encompassing the disrupted exon 2. *Bottom*. Gels use for blots above are shown stained for DNA prior to blotting. **C**. PCR screening for clones with *HP-I*-RT* allele after gene targeting with pLB-D-AseI. *Top Left.* Map of exon 6 region fro *HP-I*-RT* allele showing sites for AseI (A) and *I-SceI* (I) and position of PCR primers (arrowheads). Bottom Left. Predicted PCR product (1434 bp) and its AseI digestion products for wt allele (1086 + 348 bp) and HP-I*-RT allele (620 +466 + 348 bp). Right. Agarose gel analysis of AseI-digested PCR products of the indicated clones. L= ladder markers. Sizes (bp) are shown.

Nuclease	Sequence	Type	Size	n
gRNA1 /Cas9	ACAATGCAGACTTTGCTTTCCTTG▼GTCAGGCAGTATAATCCAAAG	P	–	–
	ACAATGCAGACTTTGCTTTCCTTG▼GTCAGGCAGTATAATCCAAAG	SD	1	2
	ACAATGCAGACTTTGCTTTCCTTG▼GTCAGGCAGTATAATCCAAAG	SD	2	1
	ACAATGCAGACTTTGCTTTCCTTG▼GTCAGGCAGTATAATCCAAAG	SD	7	1
	ACAATGCAGACTTTGCTTTCCTTG▼GTCAGGCAGTATAATCCAAAG	SD	11	1
	ACAATGCAGACTTTGCTTTCCTTG▼GTCAGGCAGTATAATCCAAAG	SD	12	1
	ACAATGCAGACTTTGCTTTCCTTG▼GTCAGGCAGTATAATCCAAAG	LD	20	1
	ACAATGCAGACTTTGCTTTCCTTG▼GTCAGGCAGTATAATCCAAAG	LD	36	1
	gaacccttctgt-784nt-CCTTG▼GTCAGGCAGTATAATCCAAAG	LD	795	1
	CAATGCAGACTTTGCTTTCCTTGC▼GTCAGGCAGTATAATCCAAAG	SI	1	1
	CAATGCAGACTTTGCTTTCCTTGG▼GTCAGGCAGTATAATCCAAAG	SI	1	2
	AATGCAGACTTTGCTTTCCTTGCT▼GTCAGGCAGTATAATCCAAAG	SI	2	1
	CAATGCAGACTTTGCTTTCCTTGi▼GTCAGGCAGTATAATCCAAAG	LI	38	1
	CAATGCAGACTTTGCTTTCCTTGi▼GTCAGGCAGTATAATCCAAAG	LI	87	1
	CAATGCAGACTTTGCTTTCCTTGi▼GTCAGGCAGTATAATCCAAAG	LI	106	1
	CAATGCAGACTTTGCTTTCCTTGi▼GTCAGGCAGTATAATCCAAAG	LI	139	3?
	CAATGCAGACTTTGCTTTCCTTGi▼GTCAGGCAGTATAATCCAAAG	LI	181	1
	CAATGCAGACTTTGCTTTCCTTGi▼GTCAGGCAGTATAATCCAAAG	LI	185	1
	AGACTTTGCTTTCCTTG-158nt-▼GTCAGGCAGTATAATCCAAAG	SD+LI	1+158	1
	GACTTTGCTTTCCTTGGGATTATA▼GTCAGGCAGTATAATCCAAAG	SD+SI	9+8	1
	ACAATGCAGACTTTGCTTTCCTTG▼GTCTGAAAGTATAATCCAAAG	SD+SI	10+8	1
gRNA2 /Cas9	ACAATGCAGACTTTGCTTTCCTTGG▼TCAGGCAGTATAATCCAAAG	P	–	–
	ACAATGCAGACTTTGCTTTCCTTGG▼TCAGGCAGTATAATCCAAAG	SD	2	1
	ACAATGCAGACTTTGCTTTCCTTGG▼CTCAGGCAGTATAATCCAAA	SI	1	1
	ACAATGCAGACTTTGCTTTCCTTGG▼-158nt-TCAGGCAGTATAA	LI	158	1
gRNA3 /Cas9	TGGCAAAACAATGATTACCCTGTT▲ATCCCTAGTCAGGCAGTATAA	P	–	–
	TGGCAAAACAATGATTACCCTGTT▲ATCCCTAGTCAGGCAGTATAA	SD	1	1
	TGGCAAAACAATGATTACCCTGTT▲ATCCCTAGTCAGGCAGTATAA	SD	9	1
	TGGCAAAACAATGATTACCCTGTT▲ATCCCTAGTCAGGCAGTATAA	SD	11	1
	GGCAAAACAATGATTACCCTGTTA▲ATCCCTAGTCAGGCAGTATAA	SI	1	3
	TGGCAAAACAATGATTACCCTGTT▲ATCCCTAGTCAGGCAGTATAA	shSD	1	2
gRNA4 /Cas9	TGGCAAAACAATGATTACCCTGT▲TATCCCTAGTCAGGCAGTATAA	P	–	–
	TGGCAAAACAATGATTACCCTGT▲TATCCCTAGTCAGGCAGTATAA	SD	1	1
	TGGCAAAACAATGATTACCCTGT▲TATCCCTAGTCAGGCAGTATAA	SD	2	1
	TGGCAAAACAATGATTACCCTGT▲TATCCCTAGTCAGGCAGTATAA	SD	3	2
	TGGCAAAACAATGATTACCCTGT▲TATCCCTAGTCAGGCAGTATAA	SD	7	1
	TGGCAAAACAATGATTACCCTGT▲TA-304nt-agaggaattttgn	LD	314	1
	GGCAAAACAATGATTACCCTGTT▲TATCCCTAGTCAGGCAGTATAA	SI	1	8
	GGCAAAACAATGATTACCCTGTC▲TATCCCTAGTCAGGCAGTATAA	SI	1	1
	GCAAAACAATGATTACCCTGTTA▲TATCCCTAGTCAGGCAGTATAA	SI	2	3
	TGGCAAAACAATGATTACCCTGT▲TATCCCTAGTCAGGCAGTATAA	shSD	7	1
	GGCAAAACAATGATTTACCCTGT▲TATCCCTAGTCAGGCAGTATAA	shSI	1	2
	nnACatCAAggcATaACCCTGTT▲TATCCCTAGTCAGGCAGTATAA	SI+pm		1
I-SceI	GGCAAAACAATGATTACCCTG-TTAT↓CCCTAGTCAGGCAGTATAA	P	–	–
	GGCAAAACAATGATTACCCTG-TTAT↓CCCTAGTCAGGCAGTATAA	SD	4	2
	GGCAAAACAATGATTACCCTG-TTAT↓CCCTAGTCAGGCAGTATAA	SD	9	1
	CAAAGACACTGG-16nt-CTG-TTAT↓CCCTAGTCAGGCAGTATAA	SD	29	2
	CAAAGACACTGG-16nt-CTG-TTAT↓CCCTAGTCAGGCAGTATAA	SD	30	2
	GGCAAAACAATGATTACCCTG-TTAT↓CTGG-145nt-CAGGCCCn	LI	153	1
	GGCAAAACAATGATTACCCTG-TTAT↓CCCAG-155nt-GAGGCCn	LI	163	1
	ATATAATTGACA-20nt-CTG-TTAT↓GGTCATCCCTAGTCAGGCA	SD+SI	34 +6	1

Figure S3. DNA sequences of exon 6 indels generated by CRISPs or *I-Sce*I. *HPRT* exon 6 DNA from 57 CRISPR-induced and ten *I-Sce*I-induced 6TG^R clones was amplified and sequenced. DNA is represented as in Figure 4, with upward and downward pointing arrowheads indicating Cas9 cleavage sites for gRNAs that recognise the coding and non-coding strands, respectively. The type, size (nt) and frequency (n) of each indel is indicated as in Figure 4 with P indicating the parental target sequence.

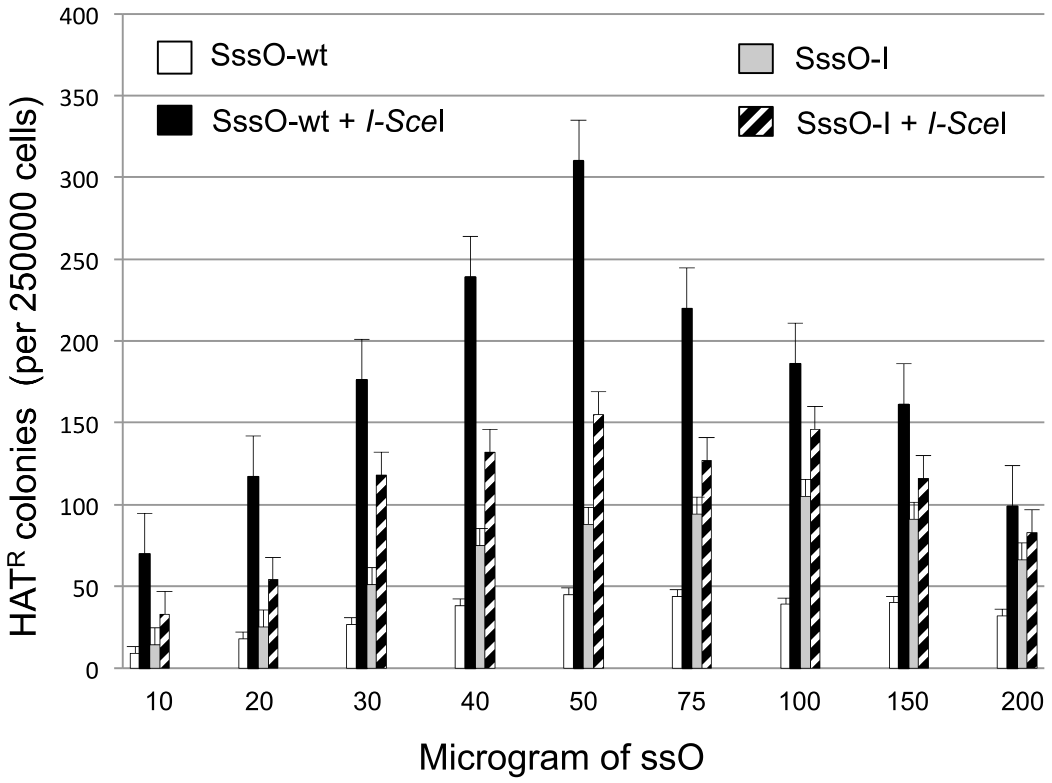

Figure S4. Effects of oligonucleotide concentration on *mHP-I*-RT* gene correction. Clone 2.1 was transfected and analysed as described in Methods and Figure 2 except that transfection was by electroporation and the indicated amounts of ssO were used.

Table S1. Summary of HT1080 cell lines used

Name (s)	Ref	Parent	Transfected DNA	Selection	Exogenous proteins	*hHPRT* allele	*mHPRT* allele	HPRT status
**HT1080 (WT)**	(1)	-	-	-	-	*WT*	-	+
**HT-HPRT:hyg**	(2)	HT1080 (WT)	pHPRThygro	hyg, 6TG	zyg	*Ex2:hyg*	-	-
**Rht14**	(3)	HT1080 (WT)	pZeoSVitTA	zeo	itTA, zeo	*WT*	-	+
**10IN-SCE.1flp1**	(3)	Rht14	pTRE-I-SceI		itTA, zeo, I-SceI	*WT*	-	+
**HTtetSCE**	TS	10IN-SCE.1flp1	pHPRThygro	hyg, 6TG	itTA, zeo, I-SceI, hyg	*Ex2:hyg*	-	-
**Clone 2.1**	TS	HTtetSCE	pSVneo-mHP-I*-RT	neo	itTA, Zeo, I-SceI, hyg, neo	*Ex2:hyg*	*mHP-I*-RT*	-
**Clones 5, 7 & 14**	TS	HPRT:hyg	pSVneo-mHP-I*-RT	neo	hyg, neo	*Ex2:hyg mHP-I*-RT*	*mHP-I*-RT*	-
**Clones 5.1, 7.1 & 14.2**	TS	m5, m7, m14	AssO-I (pCMV3xnls-I-SceI)	HAT	hyg, Neo	*Ex2:hyg*	*mHP-I-RT*	+
**Clones 5.2 & 8**	TS	HT1080 (WT)	pLB-Puro	puro	puro	*Ex6:puro*	-	-
**Clones 5.2.1 & 5.2.2**	TS	5.2	(pCMV3xnls-I-SceI)	HAT	-	*HP-I-RT*	-	+
**Clones 3B, 10B1 & 10B3**	TS	HT1080 (WT)	pLB-D-Ase	6TG	-	*HP-I*-RT*	-	-
**Clone AS5**	(4)	HT1080 (WT)	see ref	see ref	see ref	*WT*	-	+
**HT-DR-GFP-8**	(5)	HT1080 (WT)	DR-GFP	puro	puro	*WT*	-	+
**HT-SA-GFP-6**	TS (6)	Rht14	SA-GFP	puro	puro	*WT*	-	+

TS, this study; hyg, hygromycin-resistance gene or hygromycin B phosphotransferase or hygromycin B; zeo, zeocin resistance gene/protein or zeocin; Nneo, neomycin resistance gene/protein (aminoglycoside phosphotransferase) or neomycin; puro, puromycin resistance gene/protein (puromycin *N*-acetyl-transferase) or puromycin; HAT, Hypoxanthine/Aminopterin/Thymidine; 6TG, 6-thioguanine; itTA, improved tetracycline transactivator; TRE-I-SceI, I-SceI gene driven by tetracycline-response element.

Table S2. Summary of plasmids

Name	Encoded Protein/RNA	Parent plasmid(s)	Description	Ref
**pCMV3xnls-I-SceI**	I-SceI	see reference	I-SceI expression plasmid	(7)
**pSV2neo**	Neo	see reference	neo expression plasmid	(8)
**pBL-Puro/R**	Puro	see reference	Puro expression plasmid	(9)
**PGK/pDWM1**	mHPRT	see reference	Mouse *HPRT* minigene driven by PGK promoter.	(10)
**pBT/PGK-HPRT**	mHPRT	PGK/pDWM1	EcoR1 sites flank the *mHPRT* minigene cassette.	*
**pBT/PGK-HP-I-RT**	mHPRT-Q144I	pBT/PGK-HPRT	I-SceI site introduced by in vitro mutagenesis (Fig 1A).	TS
**pBT/PGK-HP-I*-RT**	truncated mHPRT	pBT/PGK-HP-I-RT	Stop codon introduced by in vitro mutagenesis (Fig 1A).	TS
**pSVneo-mHP-I*-RT**	truncated mHPRT & Neo	pBT/PGK-HP-I*-RT & pSV2neo		TS
**pBT/del-HPRT**	-	pBT/PGK-HPRT	PGK promoter deleted. Repair template dsL-wt (Fig 2A)	TS
**pBT/del-HP-I-RT**	-	pBT/PGK-HP-I-RT	PGK promoter deleted. Repair template dsL-I (Fig 2A)	TS
**pBT/del-HP-I*-RT**	-	pBT/PGK-HP-I*-RT	PGK promoter deleted. Control template dsL-I* (Fig 2A).	TS
**pLB-Puro**	Puro	pBL-Puro	HT1080 HPRT homology arms with linked I-SceI sites were amplified and cloned either side of the Puro cassette.	TS, (11)
**pJB1**	-	pLB-Puro	Puro removed from pLB-Puro by I-SceI digestion.	TS, (12)
**pJB1-Ase**	-	pJB1	AseI site and stop codon introduced by in vitro mutagenesis.	TS
**pJB2**	-	pJB1	Exon 6 region of pJB1 removed with XhoI and SphI and replaced with the equivalent wt HPRT fragment.	TS, (12)
**DR-GFP**	Puro	see reference	Reporter for intrachromsomal HR	(13)
**SA GFP**	Puro	see reference	Reporter for SSA	(14)
**pTRE-Cell Cycle**	AmCyan-hGeminin (1-100) & mCherry-hCdt1 (30-120)	-	Clontech. 631466. Geminin codons 1-100 and Cdt1 codons 30-120 fused to AmCyan and mCherry respectively.	-
**pSce-Cy-G2**	I-SceI-Cy-Gem	pTRE-Cell Cycle & pCMV3xnls-I-SceI	I-SceI-AmCyan-hGeminin (1-100) fusion protein expressed from CMV promoter.	TS
**pSce-Cy-G1**	I-SceI-Cy-Cdt	pSceI-Cy-G2 & pTRE-Cell Cycle	Geminin codons in pSceI-Cy-G2 replaced by hCdt1 codons (30-120) from pTRE-Cell Cycle.	TS
**41824**	-	pCR-Blunt II-TOPO	Vector for gRNA expression (Addgene plasmid #41824)	(15)
**41815**	hCas9	pcDNA3.3-TOPO	*S.pyogenes* Cas9 expression plasmid (Addgene plasmid #41815)	(15)
**pHPRT-gRNA-1**	HPRT-gRNA-1	41824	HPRT gRNA-1 expression plasmid	TS
**pHPRT-gRNA-2**	HPRT-gRNA-2	41824	HPRT gRNA-2 expression plasmid	TS
**pHPRT-gRNA-3**	HPRT-gRNA-3	41824	HPRT gRNA-3 expression plasmid	TS
**pHPRT-gRNA-4**	HPRT-gRNA-4	41824	HPRT gRNA-4 expression plasmid	TS

* gift from David Melton; TS = this study

Table S3 oligonucleotides

Name	Sequence (5'-3')	Purpose
**SssO-wt**	ttgacactggtaaaacaatgCAAACTTTGCTTTCCCTGgttaagcagtacagccccaaaa	mHP-I*-RT repair template
**AssO-wt**	ttttggggctgtactgcttaacCAGGGAAAGCAAAGTTTGcattgttttaccagtgtcaa	mHP-I*-RT repair template
**SssO-I**	ttgacactggtaaaacaatgATTACCCTGTTATCCCTAgttaagcagtacagccccaaaa	mHP-I*-RT repair template
**AssO-I**	ttttggggctgtactgcttaacTAGGGATAACAGGGTAATcattgttttaccagtgtcaa	mHP-I*-RT repair template
**SssO-I***	ttgacactggtaaaacaatgATTACCCTGTTATCCCTATAGaagcagtacagccccaaaa	mHP-I*-RT control template
**AssO-I***	ttttggggctgtactgcttCTATAGGGATAACAGGGTAATcattgttttaccagtgtcaa	mHP-I*-RT control template
**dsO-wt**	Annealed SssO-wt and AssO-wt	mHP-I*-RT repair template
**dsO-I**	Annealed SssO-I and AssO-I	mHP-I*-RT repair template
**dsO-I***	Annealed SssO-I* and AssO-I*	mHP-I*-RT control template

References for Tables S1 and S2

1. Rasheed, S., Nelson-Rees, W.A., Toth, E.M., Arnstein, P. and Gardner, M.B. (1974) Characterization of a newly derived human sarcoma cell line (HT-1080). *Cancer*, **33**, 1027–33.
2. Yáñez, R.J. and Porter, A.C. (1999) Gene targeting is enhanced in human cells overexpressing hRAD51. *Gene Ther.*, **6**, 1282–90.
3. Brough, R., Papanastasiou, A.M. and Porter, A.C.G. (2007) Stringent and reproducible tetracycline-regulated transgene expression by site-specific insertion at chromosomal loci with pre-characterised induction characteristics. *BMC Mol. Biol.*, **8**, 30.
4. Gravells, P., Tomita, K., Booth, A., Poznansky, J. and Porter, A.C.G. (2013) Chemical genetic analyses of quantitative changes in Cdk1 activity during the human cell cycle. *Hum. Mol. Genet.*, **22**, 2842–51.
5. Vannocci, T. (2013) The development and characterisation of a zinc finger nuclease specific for the human beta-globin gene. *PhD Thesis, Imperial College London*.
6. Hong, J.M.-L. (2011) Effects of overexpressing wild-type adn variant Rad52 on homologous recombination in human cells. *PhD Thesis, Imperial College London*.
7. Johnson, R.D., Liu, N. and Jasin, M. (1999) Mammalian XRCC2 promotes the repair of DNA double-strand breaks by homologous recombination. *Nature*, **401**, 397–9.
8. Southern, P.J. and Berg, P. (1982) Transformation of mammalian cells to antibiotic resistance with a bacterial gene under control of the SV40 early region promoter. *J. Mol. Appl. Genet.*, **1**, 327–41.
9. Yun, S., Lie-A-Cheong, C. and Porter, A.C.G. (2004) Discriminatory suppression of homologous recombination by p53. *Nucleic Acids Res.*, **32**, 6479–89.
10. Magin, T.M., McEwan, C., Milne, M., Pow, A.M., Selfridge, J. and Melton, D.W. (1992) A position- and orientation-dependent element in the first intron is required for expression of the mouse hprt gene in embryonic stem cells. *Gene*, **122**, 289–96.
11. Brustle, L.A. (2010) Developing the HPRT gene system for studying DNA break-induced gene correction. *MSc Thesis, Imperial College London*.
12. Brash, J.T. (2011) Analyses of Endonuclease- Induced Gene Modifications at the Human HPRT Locus. *MSc Thesis, Imperial College London*.
13. Pierce, A.J., Johnson, R.D., Thompson, L.H. and Jasin, M. (1999) XRCC3 promotes homology-directed repair of DNA damage in mammalian cells. *Genes Dev.*, **13**, 2633–8.
14. Stark, J.M., Pierce, A.J., Oh, J., Pastink, A. and Jasin, M. (2004) Genetic Steps of Mammalian Homologous Repair with Distinct Mutagenic Consequences. **24**, 9305–9316.
15. Mali, P., Yang, L., Esvelt, K.M., Aach, J., Guell, M., DiCarlo, J.E., Norville, J.E. and Church, G.M. (2013) RNA-guided human genome engineering via Cas9. *Science*, **339**, 823–6.
